# Contactless Real-Time Heartbeat Detection via 24 GHz Continuous-Wave Doppler Radar Using Artificial Neural Networks

**DOI:** 10.3390/s20082351

**Published:** 2020-04-21

**Authors:** Nebojša Malešević, Vladimir Petrović, Minja Belić, Christian Antfolk, Veljko Mihajlović, Milica Janković

**Affiliations:** 1 Department of Biomedical Engineering, Faculty of Engineering, Lund University, Box 118, 221 00 Lund, Sweden; christian.antfolk@bme.lth.se; 2 School of Electrical Engineering, University of Belgrade, Bulevar kralja Aleksandra 73, 11120 Belgrade, Serbia; 3 Novelic, Veljka Dugoševića 54/A3, 11000 Belgrade, Serbia; minja.belic@novelic.com (M.B.); veljko.mihajlovic@novelic.com (V.M.)

**Keywords:** artificial neural network, Doppler radar, heart rate, real-time processing

## Abstract

The measurement of human vital signs is a highly important task in a variety of environments and applications. Most notably, the electrocardiogram (ECG) is a versatile signal that could indicate various physical and psychological conditions, from signs of life to complex mental states. The measurement of the ECG relies on electrodes attached to the skin to acquire the electrical activity of the heart, which imposes certain limitations. Recently, due to the advancement of wireless technology, it has become possible to pick up heart activity in a contactless manner. Among the possible ways to wirelessly obtain information related to heart activity, methods based on mm-wave radars proved to be the most accurate in detecting the small mechanical oscillations of the human chest resulting from heartbeats. In this paper, we presented a method based on a continuous-wave Doppler radar coupled with an artificial neural network (ANN) to detect heartbeats as individual events. To keep the method computationally simple, the ANN took the raw radar signal as input, while the output was minimally processed, ensuring low latency operation (<1 s). The performance of the proposed method was evaluated with respect to an ECG reference (“ground truth”) in an experiment involving 21 healthy volunteers, who were sitting on a cushioned seat and were refrained from making excessive body movements. The results indicated that the presented approach is viable for the fast detection of individual heartbeats without heavy signal preprocessing.

## 1. Introduction

The contactless monitoring of heart rate has numerous advantages over conservative methods that use contact sensors, such as electrocardiogram (ECG) monitors, conventional photoplethysmography (PPG) sensors or piezoresistive sensors [1]. Contactless sensors offer improved mobility and obviate the need for attaching or cleaning electrodes, but also have the unique ability to be used on patients who suffer from skin irritations, painful skin damage like lacerations or burns, as well as patients who exhibit anxiety or allergic reactions to contact sensors. Furthermore, some contactless instruments, such as radar-based sensors [2], can be used for heart rate monitoring through clothes or other obstacles.

The real-time operation of heart rate monitors is required for the timely detection of potentially dangerous conditions in hospitals or in-home health care applications. The heart rate and its variability can be used for emotion, stress [3,4] or drowsiness detection [5] and real-time operation is often necessary for these applications.

In recent years, significant progress has been made in the development of radar-based heart rate monitors [6,7,8,9,10,11,12,13,14,15,16,17,18,19,20,21,22,23,24,25,26,27,28,29,30,31,32]. The potential for the production of compact low-power sensors, which are completely non-obstructive and harmless to human health, placed radar technology as one of the most promising options for contactless vital signs monitoring. Radar sensors are used for the detection of sub-millimeter movements of chest wall skin surface that occur due to heartbeats, whereas various signal processing methods are employed for heart rate extraction from discretized radar signals. Radar technology has shown not only great potential for heart rate estimation but also the potential for extracting ventricular ejection timing using nonlinear filtering methods [33].

The most frequent radar architectures used in heart rate estimation sensors are continuous-wave (CW) Doppler radars [6,7,8,9,10,11,12,13,14,15,16,17,18,19,20,21,22,23,24], frequency-modulated continuous-wave (FMCW) radars [3,25], and impulse radio ultra-wideband (IR UWB) radars [26,27,28,29,30]. CW Doppler and FMCW radars mostly outperform IR UWB radars in terms of power consumption and sensitivity [2]. The tracking of fine chest wall motion can be obtained by measuring the phase shift of the reflected signals of continuous-wave radars. The higher the frequency of the transmitted radar signal, the higher sensitivity can be obtained. While FMCW radars can detect both the absolute and relative displacement of the chest wall surface, CW Doppler radars are only capable of tracking relative displacement. This means that FMCW radars could be applied in multiple person heart rate estimation [25]. However, CW Doppler radars have a simpler hardware architecture and lower power consumption, and in single person applications, the relative displacement information obtained by the CW Doppler radar can be enough for a good heart rate estimation.

Many research groups have extensively investigated the monitoring of heart rate using CW Doppler radars. Most of the previous research was based on the experimental data monitored in studies with healthy participants lying or sitting in a controlled environment. The early published methods were based on (1) the simple filtering of heartbeat-related signals and applying a threshold to the filtered signals for extraction of heartbeat locations [6,7,8,10,12], or (2) heart rate frequency estimation using spectral analysis [9,13,15,16,17]. These approaches were hardly capable of fast and real-time performance and high-accuracy estimation at the same time. Simple band pass filtering would provide small latency, but the filtered output signals need further processing in order to automatically extract heartbeats. The robustness of these methods is hence very limited. The research in [12] showed that the error of the heart rate extraction can be drastically increased just when the subject changes their position from supine to still sitting. On the other hand, the frequency domain approaches would need a long window (5–30 s) of data for achieving sufficient frequency resolution for the detection of the heart rate harmonic. Additionally, they usually focus on high accuracy harmonic extraction and do not necessarily offer methods for distinguishing the heart rate harmonic from breathing and intermodulation harmonics. When the heart rate harmonic extraction is applied, the achieved accuracy is moderate (mean relative error of heat rate estimation around 10% [15]). Additionally, the testing set was limited to data recorded on a small number of human subjects (1 or 2, except in [12] where 10 subjects participated in the study).

However, more recent studies used data from more subjects (up to 10 participants in sitting position) and presented promising results in terms of detection accuracy. In [18], the ensemble empirical mode decomposition (EEMD) was used for the extraction of heartbeat information and in [22] the autocorrelation and frequency-time phase regression (FTPR) provided an algorithm robust to noisy conditions, but both of them used relatively long data windows for the heart rate assessment (10–15 s), which produced a large delay.

High-accuracy approaches capable of real-time operation with CW Doppler radar architectures, which achieve a relatively small delay, have recently been presented [19,20,21,23,24]. Authors in [19] and [20] used a dynamic variation of the time window for processing via the fast Fourier transform (FFT) [19] or the Wavelet transform (WT) [20]. In [21], the polyphase-basis discrete cosine transform has been used for heart rate estimation. All these methods improved heart rate detection accuracy in the frequency domain when shorter data windows were used (2–5 s). Specific heartbeat signal has been obtained using the short-time Fourier transform (STFT) analysis in [23], which was further filtered through an adaptive band pass filter for improved quality. The control of the adaptive band pass filter was done using the information extracted from the time domain analysis of the heartbeat signal on windows of 2–3 s. In [24] it has been shown that the analysis of the frequency domain only did not give satisfactory results. Therefore, the heart rate information was extracted using frequency domain analysis (window length: 3.5 s) for the coarse estimation and time domain processing using a band pass filter bank for the refinement of the results. This approach resulted in small algorithm delay (~2.5 s) and high accuracy.

Recently, new approaches based on supervised or unsupervised machine-learning algorithms [31,32] were introduced in the CW Doppler radar systems, and the first results have shown promising advantages in terms of heartbeat detection delay and source separation capabilities (robustness of heartbeat detection to respiration motion or random body motion) compared to traditional approaches. Convolutional neural networks (CNN) were applied in [29] to estimate heart rate from UWB radar signals. However, due to the lack of training data, this approach was person-specific since the CNN needed to be trained for each subject separately. To the best of our knowledge, there is no published paper that used artificial neural networks for heartbeat detection using the CW Doppler radar technology. This paper focused on the development of a system for instantaneous heart rate estimation (delay of less than 1 s from heartbeat occurrence) using a shallow artificial neural network (ANN) as a main signal processing element. Additionally, the goal was to develop a detection algorithm that was person-independent. The contribution of this work is in the development of the system for detecting individual heartbeats considering the following requirements: (1) a low-complexity time domain-based algorithm (without relying on periodic occurrences of heartbeat-related chest displacements as in the case of traditional spectral approaches), (2) suitable for real-time human presence detection, (3) calibration-free (no need for I/Q imbalance, offset compensation or the usage of any demodulation techniques) and 4) testing on a separate group of subjects from those whose heart rate signals were used in the ANN model selection and training process.

## 2. Materials and Methods

### 2.1. Basics of CW Doppler Radar Operation

**Figure 1:**
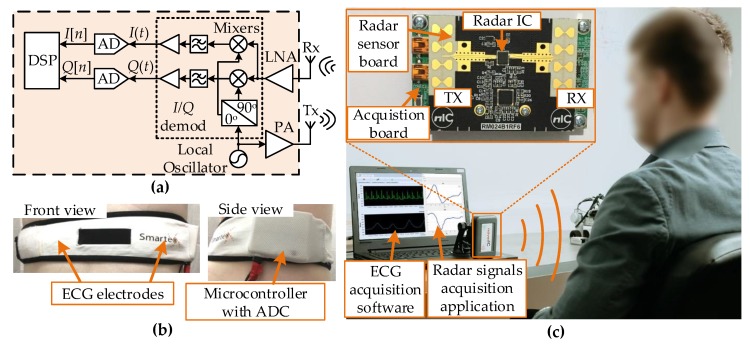
(**a**) Architecture of the quadrature Doppler radar used for the measurements; (**b**) Photographs of the Smartex Wearable Wellness System (WWS) used for the electrocardiogram (ECG) measurement; (**c**) Experiment setup.

A typical architecture of quadrature continuous-wave Doppler radar is shown in Figure 1a. The radar transmitted sinusoidal electromagnetic waves generated in the local oscillator (LO) and amplified in the power amplifier (PA). The transmitted signal reflects from the target, where the reflected signal is modulated in phase. The transmitted signal can be expressed as

(1) $$T\left(t\right)=A_{T}\cos\left(2\pi ft+\theta \left(t\right)\right),$$

where *A_T_* and *f* are the amplitude and the frequency of the transmitted signal, respectively, and *θ*(*t*) is the phase noise of the local oscillator. The received signal can be expressed as

(2) $$R\left(t\right)=A_{R}\cos\left(2\pi ft-\frac{4\pi d_{0}}{\lambda }-\frac{4\pi x\left(t\right)}{\lambda }+\theta \left(t-\frac{2d_{0}}{c}\right)\right),$$

where *A_R_*, *f* and *λ* are the amplitude, the frequency and the wavelength of the carrier signal, respectively, *c* is the speed of light, *d*_0_ is the nominal radar–target distance and *x*(*t*) is the target’s relative displacement [7]. The received signal is demodulated using a quadrature demodulator as shown in Figure 1a. The resulting signals are two baseband signals: the in-phase signal (I), which is in phase with the carrier and the quadrature signal (Q), which is phase-shifted from the carrier by 90°. These signals are expressed as [10]

(3) $$I\left(t\right)=A_{I}\cos\left(\theta_{0}+\frac{4\pi x\left(t\right)}{\lambda }+\Delta \theta \left(t\right)\right)+DC_{I},$$

(4) $$Q\left(t\right)=A_{Q}\sin\left(\theta_{0}+\frac{4\pi x\left(t\right)}{\lambda }+\Delta \theta \left(t\right)+\Delta \varphi \right)+DC_{Q},$$

where *A_I_* and *A_Q_* are amplitudes (*A_I_* ≠ *A_Q_* due to the I/Q amplitude imbalance), *DC_I_* and *DC_Q_* are DC offsets, *θ*_0_ is the constant phase shift due to the constant nominal distance *d*_0_ from (2), Δ*φ* is the phase shift due to the I/Q phase imbalance and Δ*θ*(*t*) is the total residual phase noise which can be neglected in vital signs detection applications since the distance between the target and the radar system is small [7]. Baseband signals are further digitized using analog-to-digital converters (ADCs) and processed in a digital signal processing unit.

### 2.2. Instrumentation

In this study, a CW Doppler radar with a carrier frequency of 24 GHz was used for heart rate estimation. The recording of the reference (“ground truth”) signal for the ANN training and the validation of the estimation accuracy was done using a wearable cardiorespiratory monitoring system (Smartex Wearable Wellness System (WWS), Pisa, Italy) including a single lead ECG and a piezo band as shown in Figure 1b. The ECG sensor was chosen since its accuracy was higher than other heart rate monitors such as pulse oximeters or photoplethysmographs. The system contained a microcontroller for data acquisition and Bluetooth connection for wireless data transmission to the personal computer (PC). It was a CE (Conformité Européenne) certified system. Additionally, the electrodes were connected to the skin using the wet textile fabric, which eliminated any potential irritation that could come from the sticky adhesive electrodes, particularly if applied on non-glabrous skin [34]. The sample rate for the ECG recording was 250 Hz.

The radar system used in the experiment was a Novelic Radar Module, NRM24 [24]. Figure 1c shows the radar module placed in the experimental setup. It was a DC-coupled Doppler radar sensor. The module was compact (8 × 5 × 1 cm) and portable and consisted of two stacked printed circuit boards (PCBs). The radar sensor PCB included the main part of the analog frontend: antennas, an integrated radar transceiver and a phase-locked loop integrated circuit. Antenna beamwidths (*BW*) were *BW_θ_*_, 3-dB_ = 25°, *BW_θ_*_, 6-dB_ = 33°, *BW_θ_*_, 10-dB_ = 43° (elevation) and *BW_φ_*_, 3-dB_ = 44°, *BW_φ_*_, 6-dB_ = 65°, *BW_φ_*_, 10-dB_ = 90° (azimuth), whereas the antenna gain was 12 dBi. The maximum power at the transmit antenna input was 10 dBm. The second PCB was the acquisition board, which included baseband amplifiers and filters, a power supply circuitry, an ARM Cortex-M4 based microcontroller (MCU) that integrated a multichannel 12-bit ADC and serial-to-USB converters for data transfer. The baseband filter had a cut-off frequency of 100 Hz, which was considered high enough for the vital sign detection application. The sampling rate was set to *f_S_* = 1 kHz, while the data logging on the PC was performed using a custom-made application that communicated via serial connection with the MCU.

The alignment of the datapoints from the Smartex WWS system and the radar system was done by matching timestamps in the logged data.

### 2.3. Database Recordings

**Figure 2:**
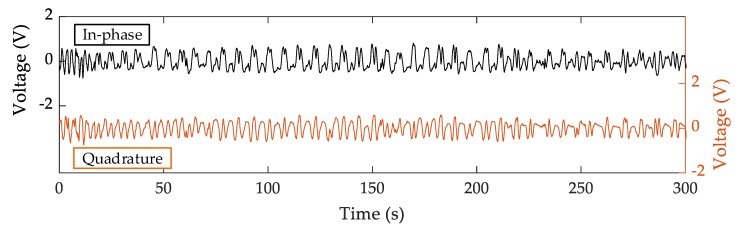
In-phase and quadrature Doppler radar signals during the measurements. The participant was breathing normally in front of the radar sensor.

The radar recordings, as well as the ECG data used as reference, were obtained from 21 healthy human volunteers who took part in the experiments (14 males and 7 females, aged 26.1 ± 5.1, with a height of 179.5 ± 11.6 cm and a weight of 74.2 ± 16.4 kg). Subjects were free of any diagnosed acute/chronic cardiac or respiratory problems, based on their self-report. Participants were acquainted with the protocol in advance and gave informed consent. The study was approved by the ethical committee of the University of Belgrade—School of Electrical Engineering, Serbia. The subjects had the wearable ECG strapped around their thorax and were instructed to sit comfortably on a cushioned seat in front of the radar sensor. The sensor was mounted on a custom stand, facing the participants at a distance of 75 cm. At this distance, the radar beam focused on the torso area, considering −3 dB beamwidths of the antenna (25° and 44°). The participants were told to breathe as they normally would in a relaxed state, without extremely deep and excessive breaths. Additionally, they were asked to refrain from excessive body and hand movements, since the rapid movements could mask the small chest wall movements that come from heartbeats and hence affect the detection. The radar signals obtained for one participant are shown in Figure 2. Three-hundred seconds of data were acquired for each subject.

**Figure 3:**
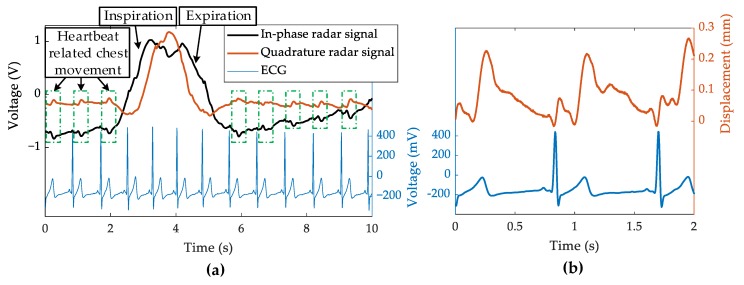
(**a**) A fragment of the recorded ECG and radar signals. It could be noted that the heartbeats acquired by the ECG amplifier are followed with slight movement patterns picked up by the radar. During the inhale−exhale (see 2–5 s) this movement pattern is distorted. (**b**) A heartbeat-related chest wall displacement obtained using the extended differentiate and cross-multiply (DACM) demodulation of the first 2 s of the radar signals.

Figure 3a shows a fragment of recorded time-aligned ECG and radar signals. There was a distinctive signal shape in the radar signals with a time delay in relation to the R wave of the ECG. The heartbeat-related disturbance in the radar signals originated from the mechanical movement of the chest wall during the heartbeat. This disturbance corresponded to the ballistocardiogram J-wave peak [35]. It can be seen that this characteristic signal shape became distorted and was reduced in the presence of breathing and movement.

Many previous works considered that the heartbeat-related displacements could be modeled as a sine wave [16], half-sine pulses [21], Gaussian pulses [22], or as an array of two consecutive pulses [24]. However, the mechanical response of the chest wall has a complex waveform that is difficult to model [33]. Figure 3b shows the heartbeat-related displacement obtained from the radar data shown in Figure 3a, using the extended differentiate and cross-multiply (DACM) demodulation algorithm described in [16]. Before the demodulation, the I/Q imbalance of the radar was measured and compensated offline like in [24]. It can be observed that the heartbeat-related displacement had a complex waveform, which further induced the complex signal shapes of the radar signals. The detector in this paper was trained with the aim to recognize these small distortions in the radar signals, without previous modeling. In order to get a reliable data set for training the detector, the signals were cropped to the period of 200 s of normal breathing (Figure 2, time interval 50–250 s).

### 2.4. Data Preprocessing

**Figure 4:**
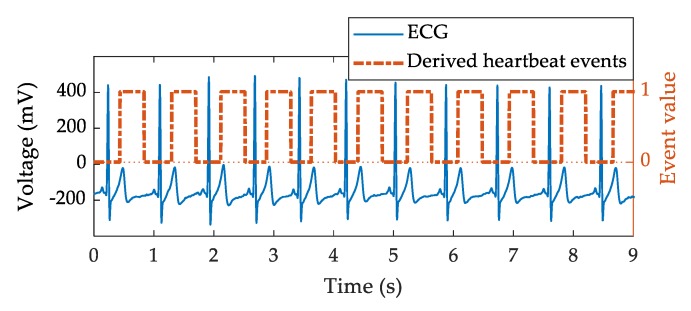
Reference ECG signal (ECG) and the binarized target signal (derived heartbeat events) where the values of “1” represent the presence of the mechanical heart displacement after the R wave, and the values of “0” represent its absence.

The detector was envisioned as a binary classifier detecting the occurrence of each heartbeat, but such implementation required the reshaping of the recorded data into an appropriate format. The continuous nature of the reference ECG signal was not suitable for this approach, so it was instead transformed into a binary on/off signal. R peaks were detected using the Pan-Tompkins algorithm [36]. The surrogate reference binary signal (“binarized target signal”) was synthesized in the form of a 400 ms pulse with 200 ms delay after each R wave in order to highlight the temporal relationship between the heart’s electrical activity (ECG) and the resulting mechanical displacement (radar signal), as shown in Figure 4. The window of 400 ms width with a latency of 200 ms after the R wave was able to cover most of the mechanical rippling observed within the radar signals. This choice is in line with a study performed on 92 healthy subjects which showed that the duration range of the R−J interval was 203–290 ms [37]. Additionally, the selection of the value of the latency parameter (200 ms) for the binarized target signal was confirmed on a recorded dataset of seven randomly selected subjects from our study. Using the simplest model that was tested in the scope of this paper (a feed-forward artificial neural network with a single hidden layer containing 10 units), a series of training and testing with a range of delays and target widths was performed. The tested delay values ranged between 0 ms to 300 ms, and the target widths were tested in the range between 300 ms and 500 ms. These values were selected based on the visual inspection of the signals, and the values that were chosen as optimal (window of 400 ms width with a latency of 200 ms after the R wave) were those that yielded the best model accuracy in terms of the percentage of detected peaks. 

The following stage in the signal preprocessing was the decimation of both the binary reference signal and the radar signal to the same sampling rate, which was a prerequisite related to the ANN employment, as each input state required a corresponding target output. To ensure the fast computation without a loss of information in the physiological range, 100 Hz was selected as the sampling rate during this computation.

The input to the ANN consisted of a 200-sample long vector, corresponding to 1 s of recording, where in-phase and quadrature branches were concatenated so that the first 100 samples in the input corresponded to the in-phase signal and the second 100 samples corresponded to the quadrature signal. Such an input was matched to the value of the reference binary signal as the target output. The choice of 1 s signal memory was based on a small-scale test conducted on a subset of recorded signals in which the memory depth varied between 200 ms and 1 s. The lower bound was sufficient to partially incorporate the mechanical wave observed due to the heartbeats and the upper bound corresponded to the interval between the subsequent heartbeats at a normal heart rate of 60 bpm. These initial test results showed that the increases of memory depth from 200 ms to 500 ms significantly increased ANN performance, while further increases resulted in only incremental gains. Nevertheless, we decided to use the deepest memory as we were not concerned about computational complexity, while using longer intervals was expected to contain more physiologically relevant data that the ANN could learn from, and not overfit to potentially insignificant signal details.

### 2.5. The Detection Algorithm

Two main approaches were taken in the design of the heartbeat detector: the classical shallow feed-forward neural networks (FF ANN) and the nonlinear autoregressive exogenous model network (NARX) as representative of the feedback-based topologies. The NARX model took as an input the current sample in the radar data stream and the previous 100 samples, together with their calculated output. The NARX topology was tested for a single hidden layer with 10 and 20 neurons, NARX 10 1 and NARX 20 1 respectively. For the classic shallow FF ANN, 4 configurations were tested: (1) a single hidden layer with 10 neurons, FF 10 1, (2) a single hidden layer with 20 neurons, FF 20 1, (3) two hidden layers with 20 and 2 neurons respectively, FF 20 2, and (4) two hidden layers with 40 and 4 neurons respectively, FF 40 4. The output layer in all the cases consisted of a single neuron with a sigmoid activation function. Activation functions for the hidden layers also varied during this topology search, including a hyperbolic tangent and log-sigmoid and linear transfer functions. Furthermore, for the FF ANNs different loss functions were tested: the mean squared normalized error (MSE), the mean squared error with regularization (MSEREG) and the sum squared error (SSE).

**Figure 5:**
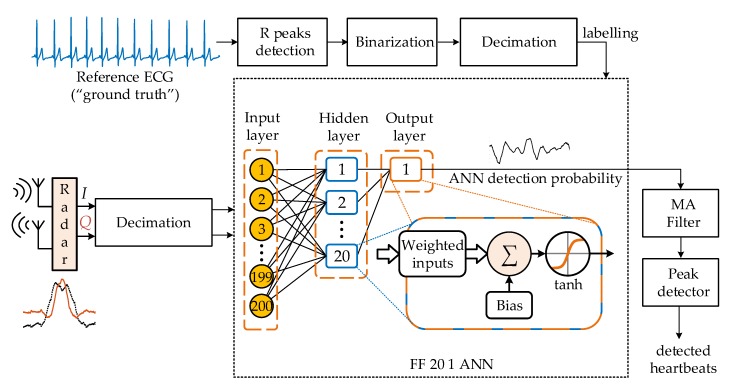
Flowchart for the proposed method for heartbeat detection based on the classical shallow feed-forward neural network with a single hidden layer with 20 neurons (FF 20 1 ANN). The artificial neural network (ANN) input is the 200-sample long vector containing resampled 100 in-phase and 100 quadrature component samples. For the FF 20 1 ANN there are 20 neurons in the single hidden layer and 1 neuron in the output layer. MA Filter—moving average filter.

As the FF ANN, containing a single hidden layer with 20 neurons, a hyperbolic tangent activation function, trained using Levenberg–Marquardt optimization and MSE loss function, outperformed all the other ANN topologies, the results presented in this paper were focused mainly on this FF 20 1 ANN topology. The whole flowchart, including the preprocessing and the detection algorithm based on the FF 20 1 ANN, is presented in Figure 5. The ANN input consisted of unprocessed in-phase and quadrature components of a Doppler radar signal (discretized and resampled *I*(*t*) and *Q*(*t*) signals from Equation (3) and Equation (4), respectively). In the FF ANN topology, each neuron in the hidden and output layers calculated a linear combination of its inputs in Equation (5):

(5) $$a_{i}^{j}=\sum_{i=1}^{N}w_{i}^{j}a_{i}^{j-1}+b_{i}^{j},$$

where *j* refers to the current layer, *N* is the number of inputs to the current layer, *w* is the weights and *b* is the corresponding biases. The output of each neuron was passed through the hyperbolic tangent function, with the exception of the output neuron which used a sigmoid function. The weights were calculated through a numerical optimization of the mean squared error loss function in Equation (6):

(6) $$MSE=\frac{1}{M}\sum_{i=1}^{M}{\left(b_{i}-a_{i}\right)}^{2},$$

where *M* is the number of data points, *b_i_* is the binarized target signal and *a_i_* is the network output. This was an iterative procedure that was set to run for a maximum of 1000 epochs or to stop early if the solution became sufficiently close to the minimum, that is, if the gradient became smaller than 10^−7^. The training would also stop if the error on the portion of the data set aside for validation (30%) failed to decrease for 6 consecutive epochs.

To remove fast noisy changes, the sequence of outputs of the ANN calculated for each sample was smoothed. This stage of the detection algorithm was implemented as a moving average filter with a width of 10 consecutive ANN outputs.

The next stage of the algorithm was the peak detection subroutine which marked local maximums of the continuous probabilities output, imposing established constraints on the minimal distance between the consecutive peaks based on the known physiological range in rest 40–120 beats/min [38,39] and their prominence (detection amplitude). When the duration of the detected inter-pulse interval (IPI) was twice as large as the previously detected IPIs (within the established constraints), a beat was interpolated as having occurred at the point in time that was the arithmetic mean between the occurrences of the current and previously detected heartbeats. The detection amplitude was defined empirically for each ANN topology on a small test sample using the error of the number of detected heartbeats as a metric. The same detection amplitude was then used for all the subjects.

### 2.6. Error Estimation and Statistical Analysis

The inter-pulse interval was calculated as the time elapsed between every two adjacent heartbeats. The classification error was determined through the percentage error in the total number of detected heartbeats and the error in the estimation of median IPIs. The similarities were also assessed between the distributions of the ANN-detected IPIs and those extracted from the reference ECG method.

**Figure 6:**
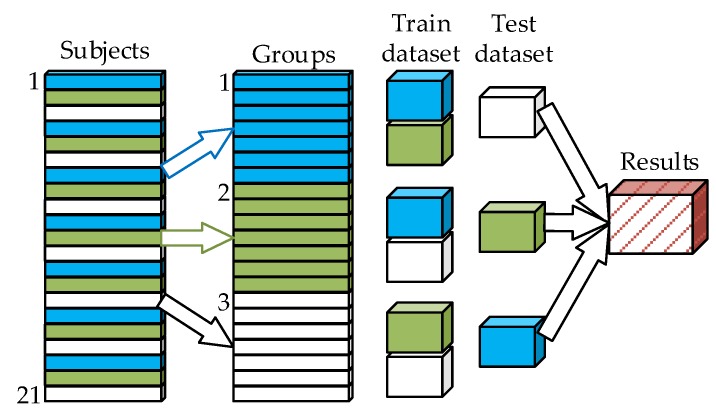
Database organization for the purpose of the ANN training and the cross-validation. The dataset was divided into three equal subsets and two subsets were used for training, while the remaining subset was used for testing. This process was repeated for all the combinations of the subsets in the training set. The three training datasets comprised 3473, 3429 and 3386 heartbeats, while the three testing datasets comprised 1671, 1715 and 1758 heartbeats.

The performance was evaluated using a three-fold cross-validation: the data were split into three equal subject groups (folds), each containing recordings acquired from 7 subjects, out of which two folds were used for training and one for testing. The training and testing were repeated three times for a different fold held out for evaluation (as shown in Figure 6).

The evaluation metrics were calculated over the set of predictions obtained on the three folds used in the test mode. A statistical analysis was performed on the results obtained via radar and those extracted from the reference ECG signal. The number of detected heartbeats and median IPI for all the recordings used as test were compared to those calculated from the reference signal. Apart from group evaluations, a statistical comparison was also performed on the level of the individual heart event detection within each of the 21-subject sets in the test mode. As in all of the statistical tests, one or more samples were found to be significantly non-normal (Lilliefors test with a 0.05 significance level),Wilcoxon signed rank test was used for statistical comparisons. 

All processing was done using the Matlab2018b (The MathWorks, Inc., Natick, Massachusetts, United States).

## 3. Results

The results obtained using six different tested ANN topologies are presented in Table 1.

The smallest error in the number of detected beats (count error—CNT error) was achieved by the FF ANN with 20 neurons in the hidden layer (2%), which was notably better than any other tested topology (12.3% < CNT Error < 34.3%). This topology also had the smallest error when the median IPIs were compared. When comparing at the level of the individual IPIs, the number of subjects whose median was significantly different from the reference was also higher than the rest, but was still relatively low.

**Figure 7:**
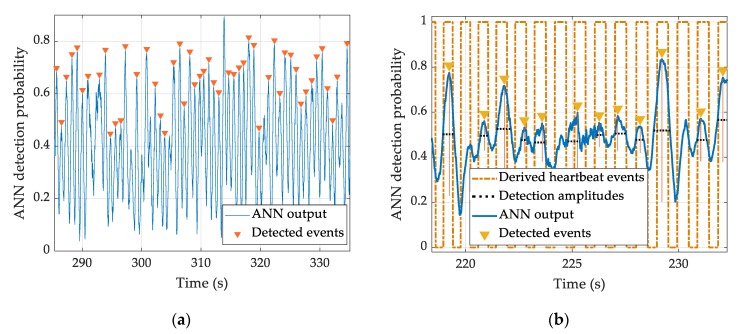
An example of the heartbeat’s method detection. Panel (a) shows the output of the FF 20 1 ANN smoothed with a moving average window, with the detected prominent peaks in a 50 s interval. Panel (b) shows the output of the FF 20 1 ANN smoothed with a moving average window on a shorter time scale plotted against the reference ECG (derived heartbeat events) for an easier visualization of the detected heart events.

Figure 7 shows an example of the output of the FF 20 1 ANN, the topology which showed the best performance, smoothed with a moving average window, with the prominent peaks detected and marked. This example shows the typical behavior and errors of the methodology. 

The error of the total number of detected heart events for the FF 20 1 ANN configuration was −2% (104 undetected beats out of a total of 5144 heartbeats extracted from the ECG). The statistical tests showed that there were no significant differences between these two methods in terms of the number of detected events (p>0.05). The difference in the medians of the IPIs calculated using the reference ECG and the FF 20 1 ANN was −2 samples (−20 ms) and was not statistically significant. When it comes to individual heart event detection, for 11 out of the 21 subjects the medians of the ANN detections were not significantly different from the reference. 

For the specific ANN that showed the best performance with the recorded database, there were 20 neurons in the first hidden layer, resulting in ~4000 multiply–accumulate operations. In the implementation on an embedded platform (Teensy 4.0 programmed in Arduino IDE) this calculation took 66 µs, which was more than enough for executing the proposed method in real time. As the calculation was done with a 100 Hz rate, there were ~10 ms in between the consecutive heart event estimations.

## 4. Discussion

The work presented in this paper is intended for the detection of individual heartbeats using a state-of-the-art mm-wave radar sensor. The radar sensor relied on the Doppler shift in the signal reflected from the objects within its field of view to detect any movements, even small ones. The real challenge of such a measurement was separating the influence from the different sources. Furthermore, smaller displacements, such as chest movements due to heart activity could be completely hidden or distorted by other physiological sources, such as breathing, talking or change of posture. Thus, the focus of this research was on the specific radar signal footprint in the time domain resulting from the heartbeats and the method by which to identify such small signal ripples. The computational tool selected for this task was shallow artificial neural networks for their high capacity for generalization, ability to be trained without prior knowledge of the signal properties and being computationally inexpensive, enabling easy implementation in an embedded or a high-level system. For the shallow ANNs that were tested, the dominant part of the computational complexity was related to the first hidden layer which performed multiplications of all the input signal samples with the weights (Figure 5).

With the aim of tracking vital signs and the presence of vehicle drivers in a contactless manner, a database of radar signals, alongside ECG and respiration, was gathered from 21 participants sitting comfortably in a cushioned chair. Up to this date, the number of participants in published papers that presented traditional or machine learning approaches for heartbeat detection was less than 12 [6,7,8,9,10,11,12,13,14,15,16,17,18,19,20,21,22,23,24,25,26,27,28,29,30,31,32], but considering our goal to obtain the realistic results of using a trained ANN on the unseen data, we acquired a larger dataset than in the previous studies. This objective imposed the strict condition of the testing of the performance on radar signals that were completely new to the ANN. Care was taken to split the data in such a manner that the signals obtained from the same person could all be found either in training or in testing (Figure 6). Although the subjects were instructed to sit quietly in the chair, some of them did substantially move their upper body and head but these recordings were nevertheless included in the database, bearing in mind the potential of neural networks to abstract over a wide variety of inputs and their robustness to noise. 

After analyzing the performance of various ANNs it was somewhat surprising to find that a relatively basic ANN outperformed more complex networks. The more complex networks were able to pick up minute details in the signals and use them to model the training set more closely at the cost of loss of generalization, while the reduced single layer network did not have such capacity to overfit. One of the conditions that favored the simpler network architectures could still be the limited database used for the training. The acquisition of yet larger amounts of data in the future could make space for more complex network architectures, such as sequential deep learning models, to further improve the results. This would, however, come at the cost of higher processing requirements.

With respect to the main idea of the ANN-based method, which was the identification of individual heartbeats, the most important metric of the ANN testing was the number of detections. Due to the lack of similar metrics in other scientific publications, the result presented in this paper of 2% undetected heartbeats could not be put into perspective with other approaches. 

As another performance evaluation metric, we calculated the time between the consecutive detections. This metric was directly compared with the IPIs from the reference ECG signal and it was shown that the difference between their medians was also not statistically significant, confirming that the method could be used to accurately track averaged heart rates in longer periods. These results were also comparable with the findings presented in [22] where the relative error between the averaged radar and ECG rates was between 0.55% and 1.97%. The method proposed by the authors [24] outperformed all of the previously published methods based on the CW Doppler radar technology regarding the mean relative error (2.07% on the dataset of ten subjects, algorithm latency ~2.5 s). The ANN-based approach presented here brings multiple advantages over other methods presented in relevant papers. The ANN method used raw radar signals without the need for any preprocessing or calibration, which can require the implementation of complex algorithms, nor prior knowledge of the signal properties and process models. The implementation of the shallow ANN within a microprocessor system was quite computationally inexpensive as it comprises only a small number of basic arithmetic operations (multiplications and additions for neural layers, while activation functions can be obtained using look-up tables). In comparison with the FFT-based approaches and the heavy use of digital filters, this feature presented a significant improvement in the computational complexity. This method is also beneficial in applications that require the fast detection of human presence, as the latency was below the width of the processing window (1 s), while to our knowledge, the shortest latency reported in relevant publications is 2.5 s [24]. This estimate of 1 s was made for the worst-case scenario of system power-up which requires the initial filling of the ANN input buffer. Once the system is up and running, with a human entering its field of view, this latency is expected to be significantly lower. Namely, due to the training procedure, the heart detections could occur in the 400 ms window that contains the latest signal samples. The smoothing by moving average filter was done on only 10 ANN outputs, which brought negligible delay. The last step of the detection chain which was peak detection required only a few extra samples to identify a local maximum.

To summarize the above contributions of our work, we listed all the relevant previously published methods for heartbeat extraction in normal breathing conditions based on the CW Doppler radar technology in Table 2. This work is the first approach that used artificial neural networks for the heartbeat detection based on the CW Doppler radar technology. The method was not person-specific (as opposed to the supervised machine learning approach applied in [32]) and we performed a more realistic scenario on 21 subjects where all the results were presented on “unseen data”. This was a fast and reliable calibration-free method, with low percentage of failed heartbeat detections and with the latency that outperformed all relevant previously published non-person-specific approaches.

As shown in Table 1, the ANN implemented in this paper showed weakness in the estimation of the IPI with high accuracies. Consequently, the utilization of the methodology for the evaluation of heart rate variability (HRV) parameters is limited. The goal of the present study was to develop a method for the fast detection of individual heartbeats; thus, all of the ANN optimizations were governed by the error of the detected heartbeats count. The HRV-related errors were not included in any of the methodology steps; therefore, it would be unrealistic to expect high accuracies compared with the methods specifically designed for the HRV estimation. In addition, the choice of the targets has a significant influence on the HRV parameters estimation. As aforementioned, the main goal was related to the robust detection of individual heartbeats, so the target window was made relatively wide (400 ms) to enable the identification of any signal waveform that was related to the mechanical displacements due to heartbeats. This wide window, except for providing more room for heartbeat detection, means that if a heartbeat was detected in any part of the target window, it would be considered as successful during the training phase. In an example, a heartbeat could be detected with the highest probability at the beginning of the target window, while the following heartbeat could be detected with the highest probability at the end, without any penalty related to the ANN performance during training. On the other hand, this kind of detection would result in 400 ms error in estimating the beat-to-beat interval.

In future work, the focus will be on increasing the HRV estimation accuracy. To achieve this goal, the error of the R–R interval estimation will be introduced as an additional loss function during the training procedure. This would inherently force the ANN to bind the maximal detection probability with a specific part of the mechanical oscillation. However, this approach would require some topological ANN changes, such as a feedback loop with the previously detected IPI and an extension of the memory depth.

In this study, there were several limitations that should be noted. All the subjects that participated in the study were young and physically fit adults. During the selected time period they were mostly sitting calmly and were instructed to restrain from prominent body movements. In this study, the chair was placed in a room with no moving objects. In a scenario which involves nonstationary objects, or recording within confined environments, such as inside a car, the performance of the system could deteriorate due to clutter and multipath effects. Given the continuous nature of the unmodulated signal, Doppler radar has no exact information on the absolute position of the observed person. Tracking vital signs of multiple people simultaneously would most likely have to be performed using modulated signals that can resolve observed targets in a space. 

The usage of a high-carrier frequency provided small dimensions of the radar system, since small antennas were used, unlike for lower frequency radar systems whose antennas usually need to be larger. An additional advantage of the high-frequency radar was its sensitivity to the small chest displacements that come from heartbeats. Since the method in this paper was using in-phase and quadrature radar signals directly, the radar sensitivity was of crucial importance for the heartbeat detection. The usage of even higher carrier frequency could improve the results, since the sensitivity to small displacements would be larger.

The radar used for this study had a relatively broad field of view, which made it susceptible to picking up clutter from the surroundings. Additionally, in applications that would require a larger distance between the radar and a patient, this broad field of view would pick up even more surrounding movements. Using an antenna with a narrower beam could improve the performance of the system in the future. It is expected that the signal-to-noise ratio (SNR) of the received signals would be smaller if the radar was placed at larger distances. This means that further tests need to be done in order to determine the influence of the SNR on the detection accuracy. Future work will also include tests of the ANN performance in cases when subjects perform natural movements, to estimate the reliability of the sensor and the method in an environment saturated with motion originating from different sources.

## 5. Conclusions

In this paper, we presented a simple and efficient contactless method for detecting individual heartbeats. The method is based on the CW Doppler radar directly coupled with an ANN stage to detect small signal ripples resulting from sub-millimeter chest movements due to heartbeats. The method has lower latency, lower computational complexity and an easier implementation on an embedded platform when compared to the traditional methodologies described in the literature, while still achieving a good heart rate estimation accuracy. With the promising results presented in this paper, we could foresee the application of the system in uses that require real-time operation, such as human detection in an industrial, automotive or clinical environment. 

## Figures and Tables

**Table 1:** Error metrics for the six different ANN topologies with reference to the ECG-derived number of heart beats and inter-pulse intervals.

Topology	CNT Error ^1^ (%)	IPI MRE ^2^ (%)	No Diff. # ^3^
FF ^4^ 10 1	−22.0	16.4	7
FF 20 1	−2.0	15.3	11
FF 20 2 1	−30.0	15.3	4
FF 40 4 1	−27.6	16.6	7
NARX ^5^ 10 1	−34.3	16.1	5
NARX 20 1	−12.3	16.4	10

^1^ Percentage error of the number of the detected heartbeats out of a total 5144 heartbeats. The negative values correspond to fewer detected heartbeats by ANN compared with the “ground truth”; ^2^ Inter-pulse interval (IPI) mean relative error; ^3^ Number of subjects with no significant difference between the medians of the estimated IPI-s and the IPI-s from the ECG reference, out of the total 21 subjects; ^4^ Feed forward ANN; ^5^ Nonlinear autoregressive exogenous model; The numbers in the topology descriptions stand for the number of units in each layer.

**Table 2:** Comparison of the methods for heartbeat extraction in normal breathing condition based on the continuous-wave (CW) Doppler radar technology.

Ref.	Radar Freq. (GHz)	*N* ^1^	TC^2^	*T*^3^ (s)	Method^4^	Unseen Data Tested^5^	*W*^6^ (s)	CF^7^	FD^8^ (%)	HR/IPIs Avg. Error^9^ (%)
[15]	2.4	5	S 80 cm	30	Multiple Signal Classification	NO	8–28	NO	-	~10
[18]	5.8	10	S 50 cm	240	Ensemble Empirical Mode Decomposition	NO	15	NO	-	3.67
[19]	5.8	4	S --	30	Time-window variation	NO	2–5	YES	-	3.3
[20]	5.8	2	S --	60	Wavelet T.	NO	3.5	YES	-	3
[21]	10.225	3	S 1.1 m	90	Discrete Cosine T.	NO	1.5 2 3	NO	-	10.4 7.6 5.1
[22]	2.4	8	S 1.5 m S 75 cm	300	Frequency–Time Phase Regression	NO	10–15	NO	-	2
[24]	24	10	S 75 cm	180	Filter bank and Chirp Z T.	NO	3.5	YES	-	1.54
[31]	24	5	S 80 cm S 30 cm T 30 cm	120	Non-negative factorization matrix	NO	8	NO	-	4.17 3.93 4.22
[32]	5.8	1	- --	600	Gamma filter	YES	15	NO	8.3	3.8
This work	24	**21**	S 75 cm	200	**ANN**	**YES**	**< 1**	**YES**	**2**	15.3

^1^ Number of subjects (N); ^2^ Test conditions (TC) during the measurement (S XX= Sitting at distance XX, T XX = Sitting and typing at distance XX); ^3^ Total measurement time (***T***) of normal breathing for each session; ^4^ Data processing approach; ^5^ Tested on data that were “unseen” in the training process; ^6^ Time window (W); ^7^ Calibration-free (CF) for I/Q imbalance, the offset compensation or usage of any demodulation techniques; ^8^ Failed detection (FD) of heartbeats; ^9^ Average Error of estimated heart rate or IPIs (HR/IPIs Avg. Error).
